# Functionalized halloysite nanotube-based carrier for intracellular delivery of antisense oligonucleotides

**DOI:** 10.1186/1556-276X-6-608

**Published:** 2011-11-28

**Authors:** Yin-Feng Shi, Zhong Tian, Yang Zhang, He-Bai Shen, Neng-Qin Jia

**Affiliations:** 1 The Education Ministry Key Laboratory of Resource Chemistry, Department of Chemistry, College of Life and Environmental Sciences, Shanghai Normal University, 100 Guilin Road, Shanghai, 200234, China

**Keywords:** halloysite nanotubes, ASODNs, cellular delivery, cytotoxicity, carrier.

## Abstract

Halloysites are cheap, abundantly available, and natural with high mechanical strength and biocompatibility. In this paper, a novel halloysite nanotube [HNT]-based gene delivery system was explored for loading and intracellular delivery of antisense oligodeoxynucleotides [ASODNs], in which functionalized HNTs [f-HNTs] were used as carriers and ASODNs as a therapeutic gene for targeting survivin. HNTs were firstly surface-modified with *γ*-aminopropyltriethoxysilane in order to facilitate further biofunctionalization. The f-HNTs and the assembled f-HNT-ASODN complexes were characterized by transmission electron microscopy [TEM], dynamic light scattering, UV-visible spectroscopy, and fluorescence spectrophotometry. The intracellular uptake and delivery efficiency of the complexes were effectively investigated by TEM, confocal microscopy, and flow cytometry. *In vitro *cytotoxicity studies of the complexes using MTT assay exhibited a significant enhancement in the cytotoxic capability. The results exhibited that f-HNT complexes could efficiently improve intracellular delivery and enhance antitumor activity of ASODNs by the nanotube carrier and could be used as novel promising vectors for gene therapy applications, which is attributed to their advantages over structures and features including a unique tubular structure, large aspect ratio, natural availability, rich functionality, good biocompatibility, and high mechanical strength.

## Introduction

Gene therapy is attractive as a clinical treatment for cancers and genetic disorders. Antisense oligodeoxynucleotides [ASODNs] are single-strand DNA molecules complementary to regions of a target gene that specifically inhibit gene expression by hybridizing the gene's mRNA [1]. Owing to their potential of selective downregulation of gene expression and modulation of gene splicing, ASODNs have attracted attention as promising therapeutic agents in the gene treatment of diseases including cancers [1-3]. Survivin, a member of the inhibitor of apoptosis gene family of proteins, is selectively overexpressed in most human cancers, but not in normal tissues [4-6]. This makes survivin a target not only for cancer diagnosis, but also for the development of novel gene therapeutic agents. ASODNs as novel anticancer agents are an area of heightened interest in the field of survivin inhibition. However, the practical application of ASODNs has faced challenges due to their susceptibility to degradation by cellular nucleases and limited intracellular uptake [7,8]. Therefore, efficient gene delivery carrier systems need to be developed to address these problems [9]. Various viral and nonviral delivery system carriers have been utilized to shuttle nucleic acids into cells, including cationic modified viruses, cationic lipids, and polymers, but each system has particular limitations [10], i.e., severe side effects (e.g., immune response and insertional mutagenesis) of viral carriers and cell toxicity of cationic carriers.

In recent years, nanomaterials as new nonviral gene carriers have attracted much attention [10,11]. Many inorganic materials including gold, carbon nanotubes, graphene oxide, and various inorganic oxide nanoparticles have been intensively studied [9-16]. Halloysites are an economically and abundantly viable clay material that can be mined from deposits [17]. Halloysite Al_2_Si_2_O_5_(OH)_4_·nH_2_O is a naturally occurring two-layered aluminosilicate, chemically similar to kaolin, which has a predominantly high-aspect-ratio hollow tubular structure in the submicrometer range and an internal diameter in the nanometer range [18]. As for most natural materials, the size of halloysite nanotubes [HNTs] generally varies from 50 to 70 nm in external diameter, a *ca*. 15-nm diameter lumen, and 0.5 to 1 μm in length. The neighboring alumina and silica layers create a packing disorder causing them to curve and roll up, forming multilayer tubes. In each HNT, the external surface is composed of siloxane (Si-O-Si) groups, whereas the internal surface consists of a gibbsite-like array of aluminol (Al-OH) groups. Even though much less studied than carbon nanotubes, due to their interesting structure and features such as unique tubular structure, large aspect ratio, cheap and abundant availability, rich functionality, good biocompatibility, and high mechanical strength, HNTs are attractive materials that show great promise in a range of applications as a nanoscale container for the encapsulation of biologically active molecules (e.g., biocides, enzymes, and drugs), as a support for immobilization of catalyst molecules, controlled drug delivery, bioimplants, and for protective coating (e.g., anticorrosion or antimolding) [19-23]. Despite these prospects, however, their utilization as biocarrier for ASODNs delivery has been less investigated so far.

In the present work, we developed a novel HNT-based drug delivery system containing ASODNs as a therapeutic gene for targeting survivin and functionalized HNTs [f-HNTs] as carriers. Herein, in order to facilitate the loading and intracellular tracking of ASODNs, f-HNTs were obtained by surface modification with *γ*-aminopropyltriethoxysilane [APTES], and fluorescein [FAM] was used to bind to ASODNs as fluorescent labeling. Furthermore, cellular uptake and delivery efficiency of the f-HNT-ASODN composites as well as cellular apoptosis induced by the ASODNs transfected with f-HNTs were investigated through confocal microscopy and flow cytometry. The results indicated that these natural, cheap, and abundantly available clay nanotubes could be used as novel vectors in the promising application of gene therapy.

## Materials and methods

All reagents used were available commercially and were of high purity grade. The survivin ASODN sequence used in the current work was 5'-CCCAGCCTTCCAGTCCCTTG and modified with fluorescently labeled on 5' end (FAM-CCCAGCCTTCCAGTCCCTTG-3'), which were obtained from Shanghai Sangon Biological Engineering Technology & Services Co., Ltd. (Shanghai, China). HNTs were purchased from NaturalNano. Inc. (Rochester, NY, USA). APTES were obtained from Sigma-Aldrich (St. Louis, MO, USA).

### Synthesis of f-HNT-ASODN complexes

**Figure 1:**
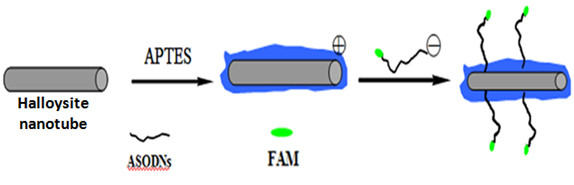
**Schematic view of f-HNT-ASODN-FAM complex preparation**.

The f-HNT-ASODN complexes were prepared as shown in Figure 1. The f-HNTs were first synthesized according to the protocols as follows [24]: briefly, 2 mL of APTES was dissolved in 25 mL of dry toluene. Approximately 0.6 g of clay powder was added, and the suspension was dispersed ultrasonically for 30 min. The suspension was then refluxed at 120°C for 20 h under constant stirring. In the refluxing system, a calcium chloride drying tube was attached to the end to ensure a dry environment. The solid phase in the resultant mixture was filtered and washed six times with fresh toluene to remove the excess organosilane, then dried overnight at 120°C. Then, ASODNs, a water soluble cationic gene drug, were bound to the anionic surfaces of the f-HNTs via electrostatic interaction. First, 30 μL of 20 μM ASODN solution and 30 μL of 1.25 mg/L f-HNTs were mixed into 1 mL water solution and stirred for 4 h at room temperature. The mixture solution was then centrifuged three times at 15, 000 rpm for 10 min. The supernatant was removed, and the deposition was dispersed in aqueous solution again with gentle sonication. Thus, free and unbound ASODNs in the f-HNT solution were removed thoroughly by repeated centrifugation, and the formed f-HNT-ASODN complexes were then resuspended.

The f-HNTs and f-HNT-ASODN complexes were characterized with transmission electron microscopy [TEM], dynamic light scattering [DLS] (Malvern Zetasizer NanoZS90, Malvern Instruments, Ltd., Worcestershire, UK), UV-visible [UV-Vis] spectrophotometry (Thermo Multiskan Spectrum, Thermo Scientific, Waltham, MA, USA), and fluorescence spectrophotometry (Varian Cary-Eclipse 500, Varian Medical Systems, Palo Alto, CA, USA).

### Cellular uptake of the f-HNT-ASODN complexes

#### Transmission electron microscopy imaging assay

HeLa cells were seeded at a density of 1 × 10^6 ^cells in a 60-mm tissue culture dish and grown overnight. The cells were incubated with the f-HNT-ASODN complexes for 6 h, and then the cells were washed thoroughly with chilled phosphate-buffered saline [PBS], centrifuged into a small pellet, and fixed with 2% glutaraldehyde in PBS (0.01 M, pH 7.4) for 120 min, and then washed three times with PBS (10 min every time). The cells were postfixed with 1% osmium tetroxide in the same buffer for 30 min, then washed three times with PBS, dehydrated through a series of alcohol concentrations (30%, 50%, 70%, 90%, 100%), embedded in Epon, and sliced to a thickness of 70 nm. Images of the sliced images were recorded at 100 kV using a Hitachi 600 TEM microscope (Hitachi High-Tech, Minato-ku, Tokyo, Japan).

#### Confocal microscopy assay

HeLa cells were seeded at 3 × 10^4 ^cells in a 35-mm Petri dish and were cultured in β-methoxyethoxymethyl ether [MEM] containing 10% fetal bovine serum [FBS] at 37°C with 5% CO_2_. After cell attachment overnight, the HeLa cells were treated with f-HNT-ASODN complexes (1.25 μg/mL), incubated for an additional 4 h in fresh media, and washed by PBS (pH 7.4) three times before confocal imaging. The cellular uptake of the f-HNT-ASODN complexes was examined by confocal laser microscopy (Carl Zeiss LSM 5 PASCAL, Oberkochen, Germany). An argon laser for FAM excitation at 488 nm was used for imaging, and an oil immersion objective (Plan Apo, SEIWA OPTICAL AMERICA INC., Santa Clara, CA, USA; magnification = 63 × 1.4) was used for cellular fluorescence imaging.

#### Flow cytometry analysis

HeLa cells were seeded in six well plates at a density of 2.5 × 10^5 ^cells/well and incubated in MEM cell culture media for 24 h at 37°C and 5% CO_2_. The cells were then incubated with f-HNT-ASODN-FAM conjugates in MEM cell culture media, and after incubation for 4 h at 37°C and 5% CO_2_, the cells were detached using trypsin, centrifuged at 1, 000 × *g *for 10 min, and analyzed using a flow cytometer (SE Diva, BD FACSVantage, Franklin Lakes, NJ, USA). A total of 1 × 10^5 ^cells were collected and analyzed for each sample. Three replicates were done for each sample. The untreated cells were used as control. The delivery efficiency was calculated as the percentage of fluorescent cells out of the total number of cells. Fluorescence was detected from the FAM labeled on ASODNs at 488-nm excitation.

#### *In vitro *cell toxicity assay of the f-HNT-ASODN complexes

HeLa cells were cultured in a MEM (Gibco, Life Technologies, Invitrogen Co., Carlsbad, CA, USA) medium supplemented with 10% FBS for 12 h at 37°C with 5% CO_2_. For *in vitro *cell toxicity assay, cells were seeded into 96 well plates at a density of 1 × 10^4 ^cells/plate and treated with ASODNs (150 nM), f-HNTs (1.25 μg/mL), and f-HNT-ASODNs, respectively. After incubation for 24, 48, and 72 h, relative cell viability was measured by standard MTT assay. In this assay, the cell viability was assessed by monitoring the enzymatic reduction of yellow tetrazolium MTT (3-(4, 5-dimethylthiazol-2-yl)-2, 5-diphenyl-tetrazolium bromide; Sigma-Aldrich, St. Louis, MO, USA) to a purple formazan, as measured at 540 nm (Thermo Multiskan spectrum, Thermo Scientific, Waltham, MA, USA). All experiments were done in six copies and illustrated as average data with error bars.

## Results and discussion

**Figure 2:**
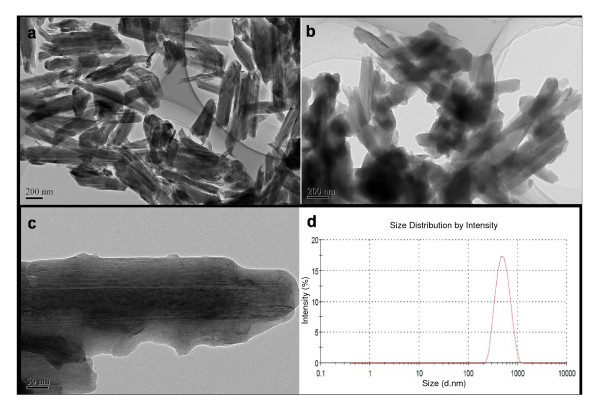
**TEM images of HNTs (a), f-HNTs (b, c), and f-HNT size distribution measured by DLS (d)**.

The morphologies and structures of HNTs and f-HNTs were first investigated. It can be seen from the TEM images (Figure 2a, b) that the HNTs and f-HNTs are hollow tubular structures with the outer diameter of approximately 70 nm, the internal diameter of approximately 15 nm, and the submicrometer range (*ca*. 500 nm) in length, whose size agreed well with the DLS analysis results(Figure 2d). TEM images of f-HNTs (Figure 2b, c) showed that there is an apparent thin layer coating on the surface of the HNTs, indicating the possible surface modification of APTES. Furthermore, zeta potential measurements (Table 1) showed that after surface modification of HNTs, the surface zeta potential value dramatically changed from -14.3 mV to +44.8 mV. The high-positive surface charges could be ascribed to the high density of the amine groups on the APTES f-HNT surface, further verifying that the negatively charged surface of HNTs was completely covered with the APTES layer. The as-synthesized f-HNTs with abundant amine groups on their surface, which provide convenient sites for further linking, make them potentially suitable for the loading and delivery of biomacromolecules.

**Table 1:** Zeta potentials of various samples dispersed in aqueous solution

Sample	Zeta potential (mV)
HNTs	-14.3
f-HNTs	44.8
ASODNs	-4.92
f-HNT-ASODNs	35.5

**Figure 3:**
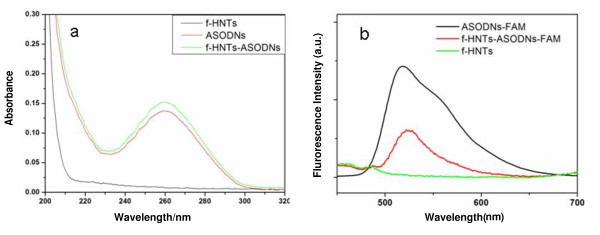
**UV-Vis absorbance (a) and fluorescence (b) spectra: f-HNTs (black), naked ASODNs (red), and f-HNT-ASODNs (green)**.

The binding of ASODNs to f-HNTs was then investigated. Zeta potential measurement, UV-Vis spectra, and photoluminescence [PL] spectra were used to observe the formation of f-HNT-ASODN complexes. After adsorption of ASODNs, the surface zeta potential of the f-HNT-based complexes decreased to a less positive value, suggesting the successful conjugation of DNA onto the f-HNT surface. Furthermore, it can be seen from spectroscopic analysis that the characteristic UV-Vis absorbance peak for ASODNs at 260 nm superimposed on the characteristic f-HNT absorption spectrum (Figure 3a). Likewise, fluorescence spectra of f-HNT-ASODN-FAM and ASODN-FAM showed a similar fluorescence peak position centering at approximately 520 nm corresponding to the characteristic emission peak of FAM molecule-conjugated ASODNs (Figure 3b). These results confirmed that ASODNs had successfully loaded onto the f-HNTs driven by electrostatic interaction.

**Figure 4:**
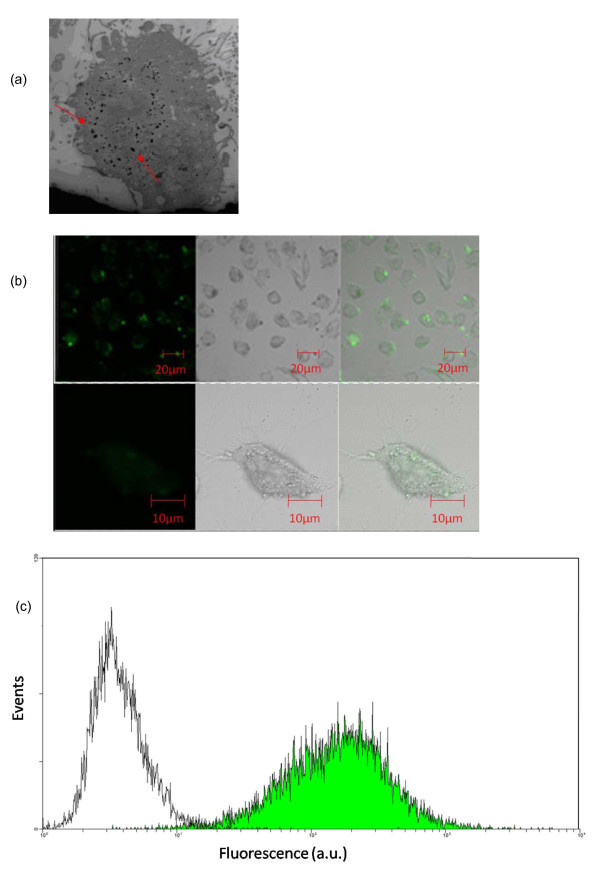
**TEM and confocal microscopy images and flow cytometry analysis of HeLa cells**. (**a**) TEM photograph of HeLa cells treated with f-HNT-ASODN-FAM complexes. (**b**) Confocal laser scanning microscopic images of the f-HNT-ASODN complexes with HeLa cell uptake (FAM fluorescence was used to label ASODNs; left), bright image (middle), and merged image (right). HeLa cells were taken after a 4-h long incubation with the f-HNT-ASODN complexes at 37°C, 5% CO_2_, and 95% relative humidity. (**c**) Flow cytometry of cells incubated with f-HNTs-ASODNs-FAM (green area) as compared with unlabeled cells (white area), demonstrating that almost each cell has been transfected. After washing, the cells were analyzed by flow cytometry. Fluorescence was detected from the FAM fluorescent material tagged on ASODNs.

To investigate the intracellular delivery ability of the f-HNT-ASODN complexes, biological TEM, confocal microscopy, and flow cytometry were applied to make qualitative and quantitative assays of the complexes' delivery into the HeLa cells. To visualize intracellular uptake of the f-HNT-based-ASODN complex, fluorescence FAM-labeled ASODNs were used for the complex formation. A TEM image of HeLa cells after incubation with the complex (Figure 4a) showed that a lot of remarkable black blots could be seen in the cytoplasm and especially around the cytoblast, indicating their effect into cells through a possible endocytosis uptake process. Confocal microscopic images (Figure 4b) also clearly showed that the f-HNT-ASODN complexes had entered into the cell cytoplasm and nucleus from the observation of the FAM fluorescent signal (green) within cells, which is in accordance with the observation done using TEM. The results suggested that the f-HNT-ASODN complexes could effectively transport into living cells.

Flow cytometry enabled the quantitative assay of delivery into the cells. HeLa cells incubated with the f-HNT-ASODN complexes for 4 h were analyzed using flow cytometry to evaluate the delivery efficiency of the complex and using FAM as fluorescence labeling. Figure 4c showed the cellular delivery efficiency of f-HNT-ASODNs estimated to be 98.69%, indicating that the f-HNTs had high intracellular delivery ability for ASODNs. Therefore, f-HNTs could be effective in transporting DNA inside the cells and could be utilized as efficient gene delivery vectors, which were mostly attributed that the stable f-HNT complex with high loading capacity could prevent DNA from enzyme degradation

**Figure 5:**
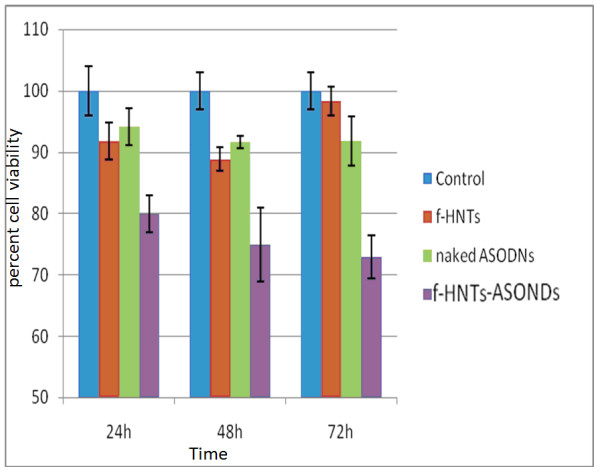
**MTT assay of treated HeLa cells**. HeLa cells treated with f-HNTs, naked ASODNs, and f-HNT-ASODNs for a period of time including 24, 48, and 72 h. Untreated cells were used as control.

In order to examine cellular apoptosis induced by the ASODNs transfected by f-HNTs, MTT assay was performed to evaluate their cytotoxicity effect on tumor cells. The HeLa cells were incubated with the f-HNT-ASODN complexes, free ASODNS, and f-HNTs for 24, 48, and 72 h, respectively. As shown in Figure 5, f-HNT-ASODN complexes displayed a significant enhancement in the cytotoxic capability compared with that of the ASODNs alone. Furthermore, the cells treated with f-HNT-ASODNs showed an increased cell apoptosis with time elongation. It was also observed that there is a minimum level of cell apoptosis upon treatment with f-HNTs, indicating that the functionalized nanotubes themselves have low cytotoxicity. Therefore, the f-HNTs could be used as a suitable carrier for therapeutic gene delivery applications due to its high surface area, efficient intracellular transporting ability, and good biocompatibility.

## Conclusions

In summary, we have prepared a novel gene delivery system with f-HNTs as carrier for loading and intracellular delivering of ASODNs. The obtained results exhibited that f-HNT-ASODN complexes could efficiently improve intracellular delivery and enhance antitumor activity of ASODNs transfected by the nanotube carrier. Therefore, with the benefits of having a unique tubular structure, large aspect ratio, abundant availability, good biocompatibility, and high mechanical strength, the HNTs could hold a great promise as a viable and inexpensive nanocarrier for biological delivery applications and gene therapy.

## Competing interests

The authors declare that they have no competing interests.

## Authors' contributions

YFS carried out the biological studies. ZT helped synthesize the material. YZ gave some help in experimental characterization. YFS drafted the manuscript. NQJ and HBS conceived the study. NQJ participated in its design and coordination and helped draft and revise the manuscript.
